# Extraction Optimization of Polysaccharides From Corn Silk and Their Antioxidant Activities *in vitro* and *in vivo*

**DOI:** 10.3389/fphar.2021.738150

**Published:** 2021-09-08

**Authors:** Liang Zhang, Yang Yang, Zhanyong Wang

**Affiliations:** College of Bioscience and Biotechnology, Shenyang Agricultural University, Shenyang, China

**Keywords:** polysaccharide, antioxidant activity, extraction, anti-aging, corn silk

## Abstract

Response surface technique was employed for improving the extraction of corn silk polysaccharides (CSP). Temperature, liquid-to-solid ratio, and per extraction time were all examined as separate factors. The optimal extraction parameters were determined by fitting experimental data to a second-order polynomial; a liquid-to-solid ratio of 21.5 ml/g, temperature equivalent to 88°C, and extraction time of 1.87 h. The experimental yield of the extracted polysaccharides following the application of these conditions was 4.33 ± 0.08% (dry weight), which fit quite well with the predicted value. CSP’s strong scavenging capabilities against hydroxyls, 1,1-diphenyl-2-picrylhydrazyl radicals, and superoxide anions along with its excellent reducing potential, were demonstrated in an *in vitro* antioxidant experiment. Meanwhile, *in vivo* testing revealed that CSP substantially enhanced glutathione peroxidase and superoxide dismutase activities. The Malondialdehyde levels in the liver and serum of aged mice also underwent a decrease. This study found that CSP has a substantial antioxidant potential *in vitro* and *in vivo*, suggesting that it might be used as an antioxidant in food and medicine.

## Introduction

With the improvement of lifestyle, people are becoming more aware of their health. Hence, the demand for health care products has manifested a noticeable rise. However, people do not only consider the efficacy, but also the source of these health care products, showing specific preference to natural health care products that are obtained from animals or plants (Necyk and Zubach-Cassano, 2017). For instance, some natural health care products are commonly used as antioxidants. Antioxidants can scavenge reactive oxygen species, which can cause oxidative stress-related illnesses (Das and Roychoudhury, 2014). Even though synthetic antioxidants can prevent or minimize the damage caused by reactive oxygen species, the demand for natural antioxidants, particularly those derived from plants, has risen in recent years owing to the former’s possible toxicological consequences (Ge et al., 2013; Shah et al., 2014). In recent years, natural polysaccharides with antioxidant activity have attracted increasing attention in natural medicine research and production (Yu et al., 2018). Corn silk is formed from stigmas, which are the yellowish thread-like strands that come from the female maize flower. It is a waste product from maize production, however, is quite plentiful (Hasanudin et al., 2012). Corn silk is a famous traditional herbal drug. In China, people have been using corn silk decoction for diabetes therapy for decades (Zhao et al., 2012). It is also used as traditional medicine in Turkey, France, and the United States (Hasanudin et al., 2012).

Carbohydrates, flavonoids, alkaloids, steroids, saponins, volatile oils, tannins, vitamins, and proteins are among the bioactive components of maize silk that have been frequently documented (Liu et al., 2011; Zhao et al., 2017). In particular, the polysaccharides extracted from corn silk (CSP) reportedly exhibited anti-fatigue (Zhao et al., 2017), anti-obesity (Chaiittianan et al., 2016), anti-diabetic effects (Zhao et al., 2012; Pan et al., 2017), antioxidant activity (Chen et al., 2013), and anti-hepatoma (Yang et al., 2014) activities.

RSM (response surface methodology) refers to a statistical strategy for the optimization of complicated operations. RSM’s major benefit is that it requires fewer experimental trials to examine various factors and their interactions (Bezerra et al., 2008). In the realm of polysaccharide extraction, RSM is widely employed (Li et al., 2017; Raza et al., 2017; Wu et al., 2017). The goal of the current investigation was to use RSM to optimize the influence of extraction temperature, per extraction duration, and liquid-to-solid ratio on CSP aqueous extraction yield and to assess the antioxidant properties of CSP. This research sheds light on how CSP can be employed as a natural antioxidant in the pharmaceutical and food sectors.

## Materials and Methods

### Chemicals and Materials

Corn silk was gathered in the Fushun suburbs, Liaoning Province, China. Sigma Chemicals Co. (St. Louis, MO, United States) provided ascorbic acid, D-galactose (D-gal), and 1,1-diphenyl-2-picrylhydrazyl (DPPH) and Sinopharm Chemical Regent Co., Ltd., Shanghai, China, furnished the rest of the reagents and solvents. All compounds used were analytical grade unless otherwise specified.

### Extraction of NCP

Corn silk was crushed and defatted in an ethanol bath at 80°C. The defatted sample (10 g) was extracted using deionized water at a certain extraction temperature, duration, and liquid-to-solid ratio after vacuum drying. The mixture was subjected to centrifugation for 15 min at 4,000 r/min to obtain the supernatant after extraction. The supernatant was combined with three portions of ethanol and stored at 4 °C for 24 h before being centrifuged again to obtain the precipitate. Centrifugation (2000 × g, 15 min) was used to extract the polysaccharide precipitate, which was subsequently deproteinated by employing a combination of proteinase and the Sevag technique (Gao et al., 2013). The CSP was obtained by lyophilizing the supernatant, which was then lyophilized to powder. The phenol–sulfuric acid technique was utilized to determine the polysaccharide content of the specimen, with D-glucose as the reference (Dubois et al., 1956).

### Design of the Experiment

The initial range of variables was defined for the extraction process and then a Box–Behnken design (BBD) was uptaken for testing the response (Y, Yield) against three independent variables: temperature (X1), liquid-to-solid ratio (X2), and per extraction time (X3). The design of the experiments was carried out using a BBD, at three levels with three factors and individual independent variables coded at three levels ranging from -1, 0, and +1. The variables were coded following Eq. 1 for statistical calculations:

$$x_{i}=\frac{X_{i}-X_{0}}{\Delta X},$$ (1)

Where *x*_*i*_ denotes a dimensionless value of an independent variable, *x*_*0*_ refers to the actual magnitude of an independent variable at the center point, *x*_*i*_ stands for the actual magnitude of an independent variable, and Δ*x* denotes the step change of the actual magnitude of the variable *i* corresponding to a variation of a unit for its dimensionless value.

The design, as indicated in Table 1, comprises 17 experimental locations with five center points to allow for pure error estimates. Each trial’s answer value was the average of duplicates.

**TABLE 1:** Box–Behnken design and results for extracted yield of CSP.

Run order	*X*_*1*_, Temperature (°C)	*X*_*2*_, Liquid-to-solid ratio (ml/g)	*X*_*3*_, Per extraction time (h)	Extracted yield of CSP (%)
1	−1 (70)	−1 (15)	0 (2)	4.01
2	1 (90)	−1 (15)	0 (2)	4.05
3	−1 (70)	1 (25)	0 (2)	3.97
4	1 (90)	1 (25)	0 (2)	4.22
5	−1 (70)	0 (20)	−1 (1)	3.81
6	1 (90)	0 (20)	−1 (1)	4.14
7	−1 (70)	0 (20)	1 (3)	3.98
8	1 (90)	0 (20)	1 (3)	4.07
9	0 (80)	−1 (15)	−1 (1)	3.92
10	0 (80)	1 (25)	−1 (1)	4.02
11	0 (80)	−1 (15)	1 (3)	3.87
12	0 (80)	1 (25)	1 (3)	3.96
13	0 (80)	0 (20)	0 (2)	4.25
14	0 (80)	0 (20)	0 (2)	4.26
15	0 (80)	0 (20)	0 (2)	4.27
16	0 (80)	0 (20)	0 (2)	4.28
17	0 (80)	0 (20)	0 (2)	4.27

To fit the second-order polynomial to the experimental data and for identification of the key components of the model, a non-linear regression approach was utilized. We described the quadratic response model by taking into account all of the linear, square, and interaction components as

$$Y=A_{0}+\overset{3}{\underset{i=3}{\sum }}A_{i}X_{i}+\overset{3}{\underset{i=3}{\sum }}A_{ii}X_{i}^{2}+\sum \overset{3}{\underset{i<j=1}{\sum }}A_{ij}X_{i}X_{j},$$ (2)

Where *A*_*0*_ stands for a constant, and *A*_*i*_, *A*_*ii*_, and *A*_*ij*_ refer to the respective coefficients of the linear, quadratic, and interactive terms, *X*_*i*_ and *X*_*j*_ represent the levels of the independent variables. To illustrate the link among the experimental and response levels of individual elements and to find the optimal circumstances, the fitted polynomial expression is profiled into surface and contour plots (Wang et al., 2015).

The experimental data were subjected to analysis by employing Design-Expert (Version 11.0.3, Stat-Ease Inc., United States) software. Three further experiments were carried out after that to ensure that the statistical experimental techniques were valid.

### Antioxidant Activity Assay *in vitro*

Hydroxyl radical scavenging activity: The potential of CSP for scavenging hydroxyl radicals was tested by making use of the method published by Smirnoff and Cumbes (1989), with minor changes. 1 ml of 8.8 mmol/L H_2_O_2_, 1 ml of 9 mmol/L salicylic acid-ethanol, 1 ml of 9 mmol/L FeSO_4_, and 1 ml of the sample solution was used in the reaction mixture. The absorbance of the combination was quantified at 510 nm following 30 min of incubation at 37°C with the positive control as ascorbic acid. The % hydroxyl radical scavenging activity was quantified making use of the following expression:

$$Hydroxyl\;radical\;scavenging\;activity\left(\%\right)=\left(1-\frac{A_{s}-A_{s0}}{A_{c}}\right)\times 100,$$ (3)

Where $A_{s}\;\mathrm{is}\;\mathrm{the}\;\mathrm{absorbance}\;\mathrm{of}\;\mathrm{the}\;\mathrm{test}\;\mathrm{sample},\;A_{c}$ denotes the absorbance of the control (deionized water instead of sample solution), and $A_{s0}$ refers to the absorbance of the sample only (deionized water in place of H_2_O_2_ solution).

Superoxide radical scavenging activity: The scavenging ability of CSP against superoxide anion radicals was measured by employing the technique published by Tian et al. (2017). The 0.4 ml sample solution was combined with Tris-HCl buffer (pH 8.2, 50 mmol/L, 4.5 ml) and allowed to incubate at 25°C for 20 min. After the addition of 0.1 ml of 2.5 mmol/L pyrogallol solution to the mixture, it was set to incubate at 25°C for 5 min. The addition of 0.1 ml of 10 mol/L HCl to the mixture resulted in the termination of the reaction. The reaction mixture’s absorbance was quantified at 325 nm. The equation outlined below was utilized for ascertaining the superoxide radical scavenging activity:

$$Superoxide\;radical\;scavenging\;activity\left(\%\right)=\left(1-\frac{A_{s}}{A_{c}}\right)\times 100,$$ (4)

Where $A_{s}$ refers to the absorbance of the samples, $A_{c}$ represents the absorbance of the control (deionized water instead of the sample).

DPPH radical scavenging activity: The assessment of DPPH radical scavenging activity was performed using Chaiklahan et al. (2013) modified technique (2013). A combination of 1 ml sample and 2 ml 0.1 mol/L DPPH was set to incubate in the dark at 25°C for 30 min. The mixture’s absorbance was estimated at 517 nm. The equation given below was employed for the calculation of the potential to scavenge the DPPH radical:

$$DPPH\;radical\;scavenging\;activity\left(\%\right)=\left(1-\frac{A_{s}}{A_{c}}\right)\times 100,$$ (5)

Where $A_{s}$ represents the absorbance of the specimen, $A_{c}$ refers to the absorbance of the control (deionized water instead of the specimen).

Fe3+ reducing antioxidant power (FRAP): FRAP was calculated using the technique given by Yildirim et al. (2001) with minor changes. 2.5 ml of 0.2 M phosphate buffer (pH 6.8), 2.5 ml of 1% (w/v) potassium ferricyanide, and 2 ml of polysaccharide sample solution made up the reaction mixture. After 30 min at 50°C, the mixture was treated with 2.5 ml of trichloroacetic acid (10% w/v) to stop the reaction and then placed into a centrifuge for 10 min at 4,000 r/min. Finally, 2.5 ml of the supernatant was added to 2.5 ml of deionized water and 0.5 ml of ferric chloride (0.1% w/v) in 2.5 ml of deionized water. At 700 nm, the absorbance was measured.

### Antioxidant Activity Assay *in vivo*

The studies were conducted with 60 Kunming mice (2-months of age, body mass BW = 20 ± 2 g), with males and females in equal numbers. The mice were acquired from Jilin University’s Pharmacology Experimental Center (Changchun, China). For 7 days, the mice were kept at a constant temperature of 25°C, humidity of 55%, and the light cycle of 12 h of light/12 h of darkness. All mice were given uninterrupted access to a normal laboratory pellet meal and water during the experiment (including acclimatization). All operations were performed by strictly adhering to the Chinese laws governing the utilization and treatment of laboratory animals.

Random assignment of the mice was made into six groups (n = 10, equal number of males and females) following a 7 day acclimation period: normal control group, D-Gal group (aging control group), ascorbic acid group (100 mg/kg BW, as positive control), and CSP groups (100, 200, and 400 mg/kg BW, as positive control). The D-Gal group received D-Gal (100 mg/kg BW) by hypodermic injection while the normal control group received an identical amount of physiological saline via gastric gavage once daily. D-Gal (100 mg/kg BW) by hypodermic injection and ascorbic acid (100 mg/kg BW) by gastric gavage was given to the ascorbic acid group once daily. CSP (as doses of 100, 200, and 400 mg/kg BW per day) and D-Gal (100 mg/kg BW per day) were given to the CSP groups through gastric gavage and hypodermic injection, respectively. For 40 days, all of the groups were fed once a day.

The mice were weighed and decapitated 24 h after the last medication treatment. To extract serum, blood specimens were taken and centrifuged at 4,000 × g for 10 min at 4°C. The liver was removed, weighed, and homogenized in 0.1 g/ml wet weight ice-cold physiological saline right away. The supernatant was recovered after centrifuging the suspension. The entire set of treatments was conducted at a temperature of 4 C.

By making use of the kits obtained from Nanjing Jiancheng Bioengineering Institute (Nanjing, China), total antioxidant capacity (TAOC), and the levels of glutathione peroxidase (GSH-Px), malondialdehyde (MDA), and superoxide dismutase (SOD) in the supernatant and blood of the liver were determined The Modified BCA Protein Assay Kit was procured from Shanghai Sangon Biological Engineering Technology and Services Co., Ltd. (Shanghai, China) to determine the protein content.

### Statistical Analysis

For statistical data interpretation, the Student’s t-test was employed. The entire set of data within the text as well as the graphics is represented as mean ± standard deviation unless otherwise specified. The computations were performed using SPSS (version 13.0).

## Results and Discussion

### BBD Analysis and Model Fitting

We used BBD to run experiments with various combinations of physical characteristics to assess the combined influence of independent factors on the response. Table 1 shows the outcomes of these trials. Under the following experimental circumstances, the highest NCP yield was recorded: extraction temperature of 80°C, the liquid-to-solid ratio of 20, and per extraction duration of 2 h. 4.28% was the highest NCP yield. Between the experimental findings, an empirical connection described by a second-order polynomial equation with interaction terms was fitted. The following is the equation derived in terms of coded factors:

$$Y=4.27+0.0887X_{1}+0.04X_{2}-0.0012X_{3}+0.0525X_{1}X_{2}-0.06X_{1}X_{3}-0.0025X_{2}X_{3}-0.073X_{1}^{2}-0.1305X_{2}^{2}-0.193X_{3}^{2}.$$ (6)

The regression coefficients were analyzing for their significance by observing their associated *p*-values, and the experimental data were examined using Pareto ANOVA (Table 2). The linear coefficients (X1 and X2), quadratic term coefficients (X12, X22, and X32), and interaction term coefficients (X1 X2 and X1 X3) were all significant (*p* < 0.05) according to the *p*-values of each model term in Table 2. The model was significant with an F-value of 34.09 and a *p*-value < 0.0001. Due to relative pure error, the lack of fit F-value of 20.71 and related *p*-value of 0.0067 was not significant. The quadratic regression model’s coefficient of determination (R^2^ = 0.9777) revealed that the model only explained 0.0223% of the overall changes. The adjusted coefficient of determination (R^2^adj = 0.949) is quite high, indicating that the model was significant, and the coefficient of variation (C.V. = 8.59%) clearly demonstrated that the experimental results were precise and reliable. Within the spectrum of experimental variables, the model was deemed adequate for prediction.

**TABLE 2:** Sequential model fitting for the yield of CSP.

Source	Statistical analysis				
	Sum of Squares	*df*	Mean Square	*F*-Value	*p*-value
Model	0.3767	9	0.0419	34.09	<0.0001
*X*_*1*_	0.0630	1	0.0630	51.32	0.0002
*X*_*2*_	0.0128	1	0.0128	10.42	0.0145
*X*_*3*_	0.0000	1	0.0000	0.0102	0.9225
*X*_*1*_ *X*_*2*_	0.0110	1	0.0110	8.98	0.0200
*X*_*1*_ *X*_*3*_	0.0144	1	0.0144	11.73	0.0111
*X*_*2*_ *X*_*3*_	0.0000	1	0.0000	0.0204	0.8906
*X*_*1*_^*2*^	0.0224	1	0.0224	18.27	0.0037
*X*_*2*_^*2*^	0.0717	1	0.0717	58.40	0.0001
*X*_*3*_^*2*^	0.1568	1	0.1568	127.73	<0.0001
Residual	0.0086	7	0.0012	–	–
Lack of Fit	0.0081	3	0.0027	20.71	0.0067
Pure Error	0.0005	4	0.0001	–	–
Cor Total	0.3853	16	–	–	–

### Optimization and Verification of the Model

**FIGURE 1:**
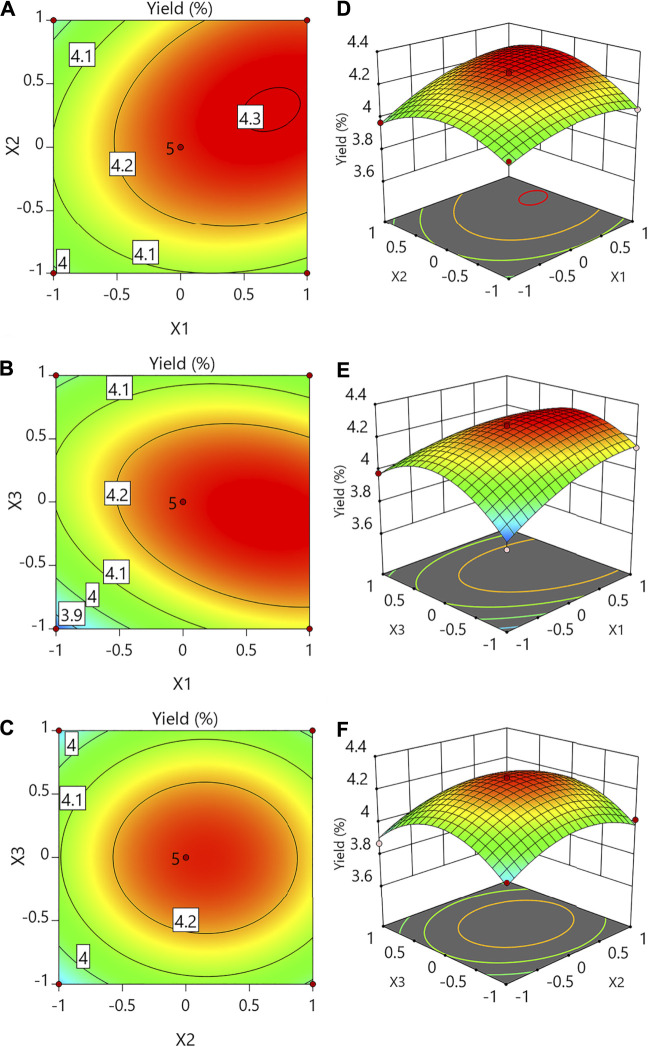
Contour plots **(A–C)** and response surface plots **(D–F)** showing the effects of variables (*X*_*1*_: temperature; *X*_*2*_: liquid-to-solid ratio; *X*_*3*_: per extraction time) on the response *Y* (yield of CSP).

To demonstrate the primary and interaction impacts of independent factors on a response variable, three-dimensional response surfaces and contour plots (Figure 1) were created. For two test variables, each curve represented an unlimited number of possibilities, while the other component was set to zero.

When the extraction duration (X3) was set to 0, the temperature (X1) and liquid-to-solid ratio (X2) had quadratic impacts on the yields, as illustrated in Figures 1A,D. The yield was calculated at various temperatures and liquid-to-solid ratios while the extraction time was kept constant. When the temperature and liquid-to-solid ratios were 88°C and 21.5 ml/g, respectively, the highest yield was obtained. Figures 1B,E depict the 3D response surface and contour plots at various temperatures (X1) and extraction times (X3) with a constant liquid-to-solid ratio (X2). When the extraction period was raised from 1 to 1.87 h, the yield rose, but it dropped after that. The yield rose when the temperature was incremented from 70°C to 88°C but did not exceed beyond 88°C. Figures 1C,F indicate that while the temperature (X1) was kept constant at level 0, the liquid-to-solid ratio (X2) and per extraction time (X3) had quadratic impacts on the response yield. As the liquid-to-solid ratio underwent an increase and the pre-extraction time also demonstrated an increase, there was first an increase in the yield which dropped later. When the liquid-to-solid ratio and per extraction time were 21.5 ml/g and 1.87 h, respectively, the highest yield was obtained.

The anticipated yield values (4.31%) were reached under the following conditions: temperature of 88°C, the liquid-to-solid ratio of 21.5 ml/g, and per extraction time of 1.87 h, according to the plots. The verified experimental yield was 4.33 ± 0.08% at the above optimum circumstances. The mean value calculated from real trials verified the RSM model and suggested that it was acceptable for extraction when compared to the projected value.

### Antioxidant Activities of CSP *in vitro*

**FIGURE 2:**
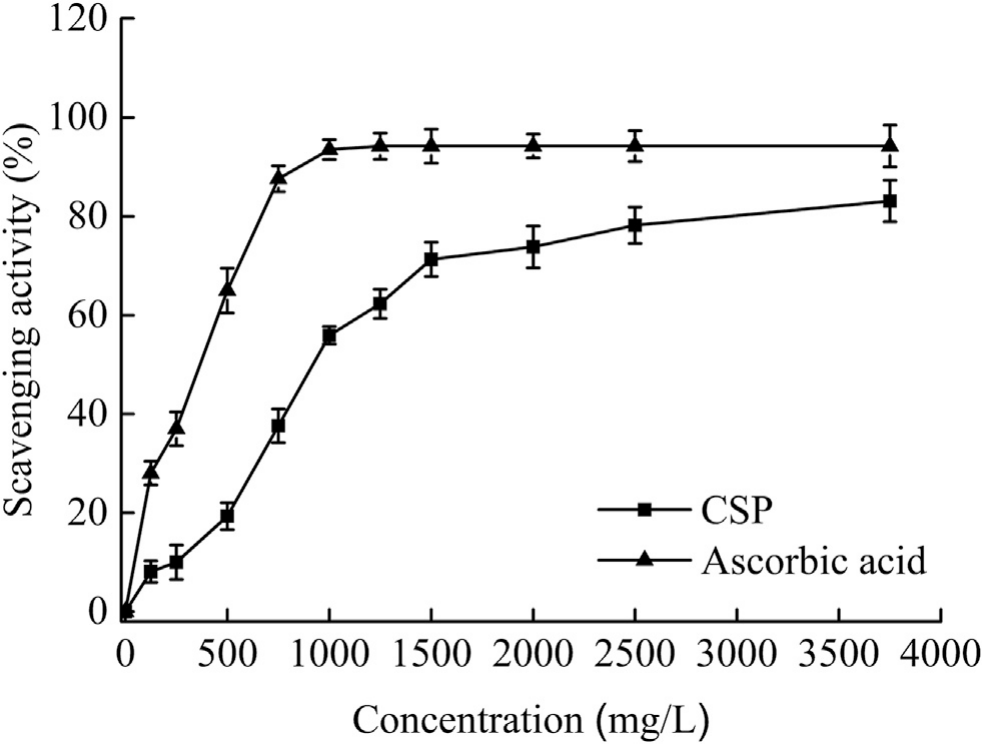
Scavenging effects on hydroxyl radicals of CSP.

The Fenton reaction or the iron-catalyzed Haber–Weiss reaction create hydroxyl radicals. They are so reactive that they can harm most biomolecules, although antioxidants can minimize their effects (Li et al., 2012). Figure 2 depicts that the scavenging influence of CSP on hydroxyl radicals increased across the concentration range examined, but the scavenging effect of ascorbic acid on hydroxyl radicals was significantly greater (*p* < 0.05). CSP’s EC_50_ against hydroxyl radicals was 918.2 mg/L, which was much greater than ascorbic acid’s (365.6 mg/L). CSP has an EC_50_ similar to EUPS-2 (CSP extracted using an enzymolysis–ultrasonic technique) (Chen et al., 2014). There are two antioxidant processes for hydroxyl radicals: inhibition of hydroxyl radical production and direct scavenging of hydroxyl radicals (Ueda et al., 1996). The interaction of hydrogen with radicals and the subsequent cessation of the radical chain reaction was also described as part of the hydroxyl radical scavenging process by polysaccharides (Shi et al., 2013). As a result, the mechanism by which CSP scavenges hydroxyl radicals has to be investigated further.

**FIGURE 3:**
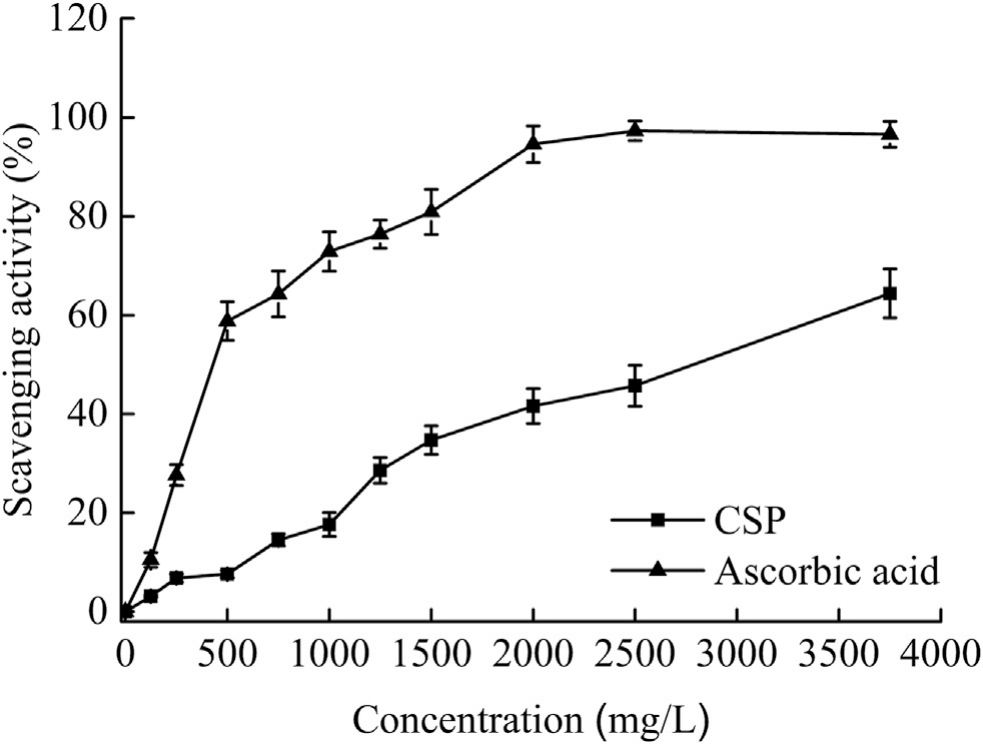
Scavenging effects on superoxide anion radicals of CSP.

A specific number of superoxide anion radicals are found within the human body. When superoxide anion radicals mix with hydroxyl radicals, cell DNA is damaged, and the human body’s function is compromised (Tian et al., 2017). Figure 3 indicates that when the concentration of CSP rose, the rate of elimination of superoxide anion radicals increased, and the scavenging rate was proportional to the CSP concentration within the test dose range. The R^2^ coefficient was 0.9867. Within the test dose range, CSP scavenging rates were lower than ascorbic acid scavenging rates. CSP had a greater EC_50_ (2,775.6 mg/L) than ascorbic acid (402.3 mg/L). Although superoxide is a mild oxidant, it can deteriorate over time and produce additional reactive oxygen entities for instance singlet oxygen and hydroxyl radicals, which can lead to lipid peroxidation (Qi et al., 2005). Pathological events, such as arthritis and Alzheimer’s disease, can also be caused by superoxide (Sun et al., 2010). As a result, superoxide anion scavenging is critical to antioxidant activity. Our findings show that CSP has a significant scavenging effect on superoxide radicals.

**FIGURE 4:**
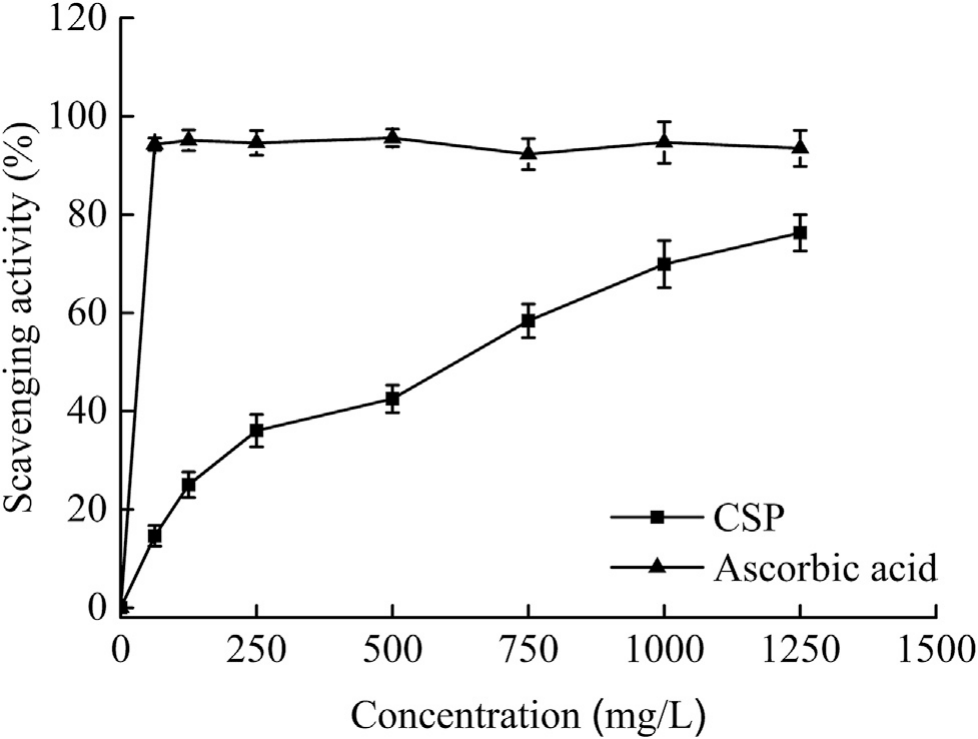
Scavenging effects on DPPH radicals of CSP.

As astable radical, DPPH may be used to test an antioxidant’s scavenging capability. As a result, it’s been frequently utilized to assess the radical-scavenging abilities of naturally occurring chemicals (Leong and Shui, 2002). Figure 4 depicts the DPPH radical scavenging effects of CSP. In a concentration-dependent way, CSP scavenged the DPPH radical. Within the test dose range, CSP and ascorbic acid had their highest scavenging activities at 3,750 and 250 mg/L, respectively. CSP’s scavenging ability against DPPH radical had an EC_50_ of 603.2 mg/L, which was greater than ascorbic acid’s (34.9 mg/L). The typical absorption maxima of DPPH in alcohol is 517 nm. When a hydrogen-atom-donating antioxidant is introduced to DPPH, the DPPH radical is scavenged, and the absorbance at 517 nm vanishes (Marinova and Yanishlieva, 1997). These findings suggest that CSP scavenges DPPH by acting as an electron or hydrogen donor.

**FIGURE 5:**
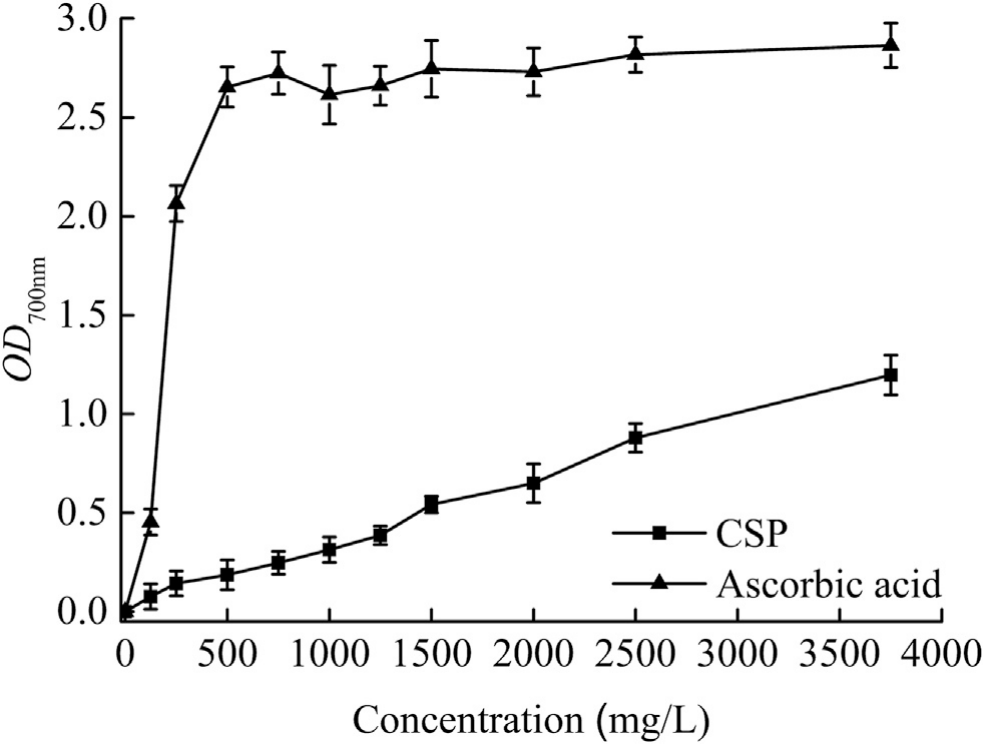
Fe^3+^ reducing antioxidant power of CSP.

A compound’s reducing capacity is an important indication of its potential antioxidant action (Kallithraka et al., 2001). The FRAP of CSP rose with the rise in concentration, but stayed low in comparison to ascorbic acid, as seen in Figure 5. The existence of reductones, which serve as antioxidants by disintegrating the free-radical chain after a donation of a hydrogen atom, was typically linked to the reducing capabilities (Amarowicz et al., 2004). The antioxidant activity of a substance can be determined by its reducing capacity (Meir et al., 1995). The current findings on CSP’s FRAP suggest that it has an impact on its antioxidant activity.

### Antioxidant Activity of CSP *in vivo*

Tables 3 and 4 demonstrate the influence of CSP on SOD, CAT, GSH-Px, TAOC, and MDA in the sera and livers of aged mice. As opposed to the normal control group, the activities of antioxidant enzymes (SOD and GSH-Px) and the amount of TAOC in the sera and livers of the aging control group substantially reduced (*p* <0.01; GSH-Px in livers *p* <0.05), whereas MDA underwent a significant increase (*p* <0.01). These findings suggest that the aging mice model was successfully developed. In all dosages examined, CSP considerably enhanced (*p* <0.01) SOD activity and significantly lowered (*p* <0.01) MDA levels as opposed to the aging control. The extract significantly enhanced (*p* <0.01) GSH-Px activity and TAOC level in a dose-dependent fashion.

**TABLE 3:** Effects of CSP on the activities of SOD, GSH-Px, TAOC and MDA levels in serums in aging mice.

Test Groups	SOD (U/mL)	GSH-Px (U/mL)	TAOC (U/mL)	MDA (nmol/ml)
Normal control	277.82±24.56	158.33±16.68	16.52±1.62	11.47±0.78
Aging control	194.33±17.16^d^	144.75±11.52^c^	9.69±1.68^d^	15.78±1.06^d^
Ascorbic acid (100 mg/kg positive control)	266.98±24.53^b^	177.83±12.35^b^	15.13±0.34^b^	11.14±0.85^b^
CSP (100 mg/kg)	232.47±17.96^b^	146.57±8.69	10.25±0.82	13.12±1.05^b^
CSP (200 mg/kg)	257.38±21.49^b^	157.68±11.63^a^	12.46±1.74^a^	12.88±0.97^b^
CSP (400 mg/kg)	276.86±17.68^b^	161.32±13.51^a^	15.57±2.15^b^	12.79±1.15^b^

Values are mean ± SD.

a *p* <0.05.

b *p* <0.01 vs Aging control.

c *p* < 0.05.

d *p* < 0.01 vs Normal control.

**TABLE 4:** Effects of CSP on the activities of SOD, GSH-Px, TAOC and MDA levels in livers in aging mice.

Groups	SOD (U/mg protein)	GSH-Px (U/mg protein)	TAOC (U/mg protein)	MDA (nmol/mg protein)
Normal control	354.65±28.56	141.25±17.35	6.34±0.14	2.67±0.24
Aging control	188.68±24.59^c^	87.69±11.72^c^	3.24±0.25^c^	5.75±0.33^c^
Ascorbic acid (100 mg/kg positive control)	377.35±31.25^b^	149.47±10.56^b^	5.27±0.24^b^	2.94±0.24^b^
CSP (100 mg/kg)	261.25±25.69^b^	98.47±8.52^a^	3.95±0.24^a^	4.27±0.31^b^
CSP (200 mg/kg)	323.25±25.17^b^	124.54±9.65^b^	4.73±0.18^b^	4.01±0.36^b^
CSP (400 mg/kg)	373.14±19.35^b^	147.25±15.38^b^	5.74±0.37^b^	3.76±0.32^b^

Values are mean ± SD.

a *p* <0.05.

b *p* <0.01 vs Aging control.

c *p* < 0.01 vs Normal control.

Antioxidant enzymes are biological macromolecules initial line of defense against oxidative damage. SOD and GSH-Px are key antioxidant defense systems enzymatically (Zhang et al., 2003). SOD, by catalyzing superoxide elimination, defends against oxygen free radicals, thereby damaging membranes and biological entities, whereas GSH-Px catalyzes the reduction of H_2_O_2_ to O_2_ and H_2_O at the expense of GSH (Lv et al., 2007). The non-enzymatic antioxidant defense mechanism in animals can be reflected in the amount of TAOC (Zhang et al., 2003). In the current study, CSP enhanced TAOC and elevated SOD and GSH-Px activity in aged mice. The findings suggest that CSP’s antioxidative mechanism is partially due to its effects on antioxidant enzymes as well as non-enzymatic systems.

As a primary product of lipid peroxidation, MDA, functions as an oxidative damage indicator and aging assessment index (Shen et al., 2018). It’s one of the most common indicators of endogenous lipid peroxidation. MDA production rises with age, indicating that peroxidative damage rises as well (Rotruck et al., 1973). Because of the reduction in MDA generation in aged mice, our findings suggest that CSP was effective in inhibiting lipid peroxidation. Earlier investigations (Zhong et al., 2013; Govindan et al., 2016) have also depicted that polysaccharides significantly lower MDA levels when compared against the control group.

## Conclusion

The individual and interactive impact of process variables (temperature, liquid-to-solid ratio, and per extraction time) on CSP yield were effectively optimized and evaluated using RSM. The findings revealed that process factors had a substantial impact on CSP maximum yield. The experimental values for the polysaccharide yield were quite near to the predicted yield under these optimized circumstances. *In vitro* antioxidant activities of hydroxyl radicals, superoxide anion radicals, DPPH, and FRAP revealed that CSP possesses antioxidant properties that increase with increasing polysaccharide content. CSP substantially incremented the activity of antioxidant enzymes and dramatically decreased the concentration of MDA within the liver and serum of aging mice in anti-aging tests. CSP substantially reduces aging-related damage by replenishing antioxidant enzymes and lowering lipid peroxide, according to the study. As a result, CSP demonstrates promising potential to be incorporated into the diet as a functional natural food to prevent and treat aging-related damage.

## Data Availability

The raw data supporting the conclusions of this article will be made available by the authors, without undue reservation.
